# Design and reporting of interventional clinical trials

**DOI:** 10.1038/s41467-022-28548-6

**Published:** 2022-02-17

## Abstract

Ruth Plummer is Professor of Experimental Cancer Medicine, Newcastle University, and an honorary consultant medical oncologist in Newcastle Hospitals NHS Foundation Trust. She directs the Sir Bobby Robson Cancer Trials Research Centre and leads the Newcastle Experimental Cancer Medicine Centre and CRUK Newcastle Cancer Centre. She has taken multiple agents targeting DDR into the clinic, including the first-in-human PARP and ATR inhibitors. In addition, she has an active clinical practice treating skin cancer, both in the advanced and adjuvant settings and with an associated clinical trials portfolio including both early and later phase trials. In this interview for Nature Communications, Ruth Plummer shares her knowledge about the basic principles for the design of clinical trials and how they should be reported.

**Figure:**
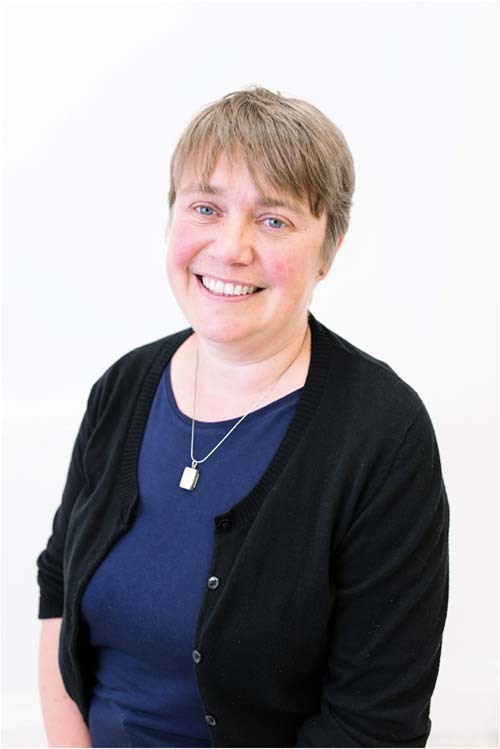
Ruth Plummer

Clinical study protocols are the foundation of clinical trials. What information should an ideal protocol contain and why should the trial be conducted in strict adherence to it?

The clinical trial protocol is the critical guidance for the staff treating the patient who agrees to go on the study—this includes the clinical research staff and also any staff who are involved in the patients’ care, especially if they have an emergency admission during participation in a study. For this reason, the protocol needs to contain an outline of the purpose of the study, what the treatments given are, and most importantly the key safety data about any experimental treatment, with brief summaries of likely toxicities or side effects and guidance on how to manage them. For randomized and blinded studies the protocol also needs to contain information on the process to break this in an emergency.

In addition, the protocol should have a clear schedule of events so clinical research staff at all sites can easily ensure patients are given the same treatment doses and regimen, and the same research samples are collected. Strict adherence to the protocol is important as it will have been designed and reviewed by the regulators with patient safety in mind, and also to ensure the scientific integrity of the research questions being asked for the potential benefit of the wider patient community.

One of the key aspects of the protocol is sample size estimation. How important is sample size estimation for a clinical trial? What are the effects of sample size over-estimation or under-estimation on the outcome of a trial?

It is key for the development of any protocol to have expert statistical input and any clinical investigator designing a trial protocol works closely with their statistical colleagues to make the best estimation of sample size. This is important so that we can have the best chance of answering the research question—usually, whether a new treatment is more effective than an existing one—meaning we determine if we are offering the best treatment options to our patients as standard of care. Over-estimating the number of participants that need to be recruited may mean the trial will take longer to complete, and if treatment is not effective more patients will be exposed to it. Under-estimation of the number of participants risks that the outputs of the trial do not reach statistical significance, so cannot be said not to have happened by chance and may mean an effective treatment is rejected, or another trial needs to be done.

What are the different phases in clinical trials? What is the purpose of each of them?

Early phase trials are safety studies, and then late-phase trials are the randomized trials that are generally required before a new treatment is considered for licensing and wider use.

Phase I studies have the primary endpoints of safety and recommended phase II dose—so these trials look to establish the best dose of a drug or combination for further clinical testing. These are also the studies that explore for the first time the clinical pharmacology of a new agent in humans. Phase II studies are traditionally the first efficacy studies and inform the design of the pre-registration phase III study. Therefore, these phase III studies are the pivotal trials that allow a potential new treatment to be considered by the regulators as a standard treatment that would be widely available and recommended. Phase IV studies are carried out post-marketing, often for safety surveillance.

What is the difference between primary and secondary outcomes? Should a clinical trial publication include all the pre-specified outcome measures and at the pre-specified follow-up time? What are the drawbacks of not doing so?

The primary outcome of any study is the key measure to be evaluated by the trial, and the one around which all the sample size and statistical analyses are based. Secondary outcomes follow on from this and are frequently linked. For example, the primary outcome in a cancer trial might be overall survival with a secondary outcome of progression-free survival or time to next treatment being needed. In the early phase safety studies, which are the majority of my clinical practice, primary outcomes are typically the toxicity data reported or recommended phase II dose, with secondary outcomes of drug pharmacokinetics, and response.

Any clinical trial publication does need to report the primary outcome, as this will be the statistically valid output, and also the primary question being asked by the study and what was explained to patients when they consented to take part. Ideally, a trial publication should include all pre-specified endpoints so that for the design of ongoing studies or treatments researchers have all the information available and can make the best-informed decisions. However, there are situations where either in the case of a clear benefit to patients or a safety signal/potential harm one would want these data published ahead of the completion of all pre-specified analyses.

What is the value of ad hoc analyses of clinical trials? How can these be properly reported in a scientific publication?

Ad hoc analyses can bring out unexpected findings—such as patient factors indicating who might benefit most from a new treatment, or may identify factors that predict side effects. These can be reported within a scientific publication, and to my mind certainly should be as they can inform the next studies and refinement of best treatments. It needs to be clear that these are ad hoc, and not pre-planned as the statistical validity is different. These analyses are typically used to inform the design of further clinical trials, and the regulators would rarely use analysis of data that was not pre-planned or specified to recommend clinical practice changes although clear safety signals identified in any such analysis should be adopted.

Recently, regulators and publishers have made the inclusion of a data-sharing plan in the protocol and a data-sharing statement in the scientific publication important requirements for clinical trials. What is the reason behind this? What information should these documents contain?

The advantages of data sharing are huge. It helps ensure that clinical trial data is not only published and available to the scientific and clinical communities (particularly important for negative studies to avoid these being repeated) but also can be accessed by external researchers—so that conclusions can be checked. Patients in studies are “gifting” their time and samples to help with research, and we have a duty to ensure this generosity is put to the best use, so sharing data and information on samples available is key to allowing this. All data sharing plans make it clear that the data is curated and no patient identifiable data will be shared, and this assurance is a key part of the trial consent process. Data sharing documents should contain both the “processed” data tables and ideally raw data—such as pharmacokinetic and biomarker data—in a form that others can analyze.

